# The Relative Contribution of Root Morphology and Arbuscular Mycorrhizal Fungal Colonization on Phosphorus Uptake in Rice/Soybean Intercropping Under Dry Cultivation

**DOI:** 10.3390/plants14010106

**Published:** 2025-01-02

**Authors:** Huimin Ma, Hongcheng Zhang, Qian Gao, Shilin Li, Yuanyuan Yu, Jiaying Ma, Congcong Zheng, Meng Cui, Zhihai Wu, Hualiang Zhang

**Affiliations:** 1 Faculty of Agronomy, Jilin Agricultural University, Changchun 130118, China; 2 INRAE, UMR ISPA, 33140 Villenave d’Ornon, France; 3 State Key Laboratory of Vegetation and Environmental Change, Institute of Botany, Chinese Academy of Sciences, Beijing 100093, China; 4 Zhejiang Provincial Key Laboratory of Agricultural Resources and Environment, Institute of Soil and Water Resources and Environmental Science, College of Environmental and Resource Sciences, Zhejiang University, Hangzhou 310058, China

**Keywords:** rice/soybean intercropping, P uptake, root morphology, AMF colonization

## Abstract

Intercropping has the potential to improve phosphorus (P) uptake and crop growth, but the potential benefits and relative contributions of root morphology and arbuscular mycorrhizal fungi (AMF) colonization are largely unknown for the intercropping of rice and soybean under dry cultivation. Both field and pot experiments were conducted with dry-cultivated rice (*Oryza sativa* L.) and soybean (*Glycine max* L. Merr.) grown alone or intercropped under two P levels. Two root separation modes between rice and soybean were employed to explore the contribution of AMF association and root plasticity on P uptake in intercrops. The results showed that rice/soybean intercropping resulted in a notable increase in the total biomass and yield compared to monoculture in the field. In the potted experiment, compared to the plastic root separation treatment (PS), the no root separation treatment (NS) increased the total biomass and P uptake by 9.4% and 19.9%, irrespective of the P levels. This was primarily attributable to a considerable enhancement in biomass and phosphorus uptake in soybean by 40.4% and 49.7%, which offset a slight decline in the rice of NS compared to PS by 26.8% and 18.0%, respectively. The results of random forest analysis indicate that the P uptake by the dominant species, soybean, was mainly contributed by root morphology, while rice was more dependent on AMF colonization in the intercropping system. Therefore, dry-cultivated rice/soybean intercropping enhances P uptake and productivity by leveraging complementary belowground strategies, with soybean benefiting primarily from root morphological adjustments and rice relying more on arbuscular mycorrhizal fungi colonization.

## 1. Introduction

Phosphorus (P) is one of the most essential nutrient elements for crop growth [1]. In response to soil P deficiency, farmers often apply a large quantity of P fertilizer for high yields, which leads to reduced P use efficiency accompanied with severe environmental pollution [2]. Thus, the enhanced P uptake and utilization efficiency of crops are necessary for the sustainability of intensive agriculture [3]. Intercropping is an effective agronomic approach to improve soil P uptake and utilization [4]. Among all the intercropping combinations, cereal/legume intercropping systems have a longer development history. Many studies have shown that cereal/legume intercropping has higher yields and P uptake compared to the corresponding monoculture crops [5,6,7]. Rice (*Oryza sativa* L.) is a staple food in both China and throughout the world [8]. Dry cultivation is a new rice crop mode used to alleviate water shortages and develop water-saving agriculture [9]. However, the growth of dry-cultivated rice is often limited by the lack of soil P [10]. In addition, legumes can mobilize insoluble P through root exudations and rhizosphere soil microbial communities, thereby promoting the P uptake of associated intercropping cereals [11,12]. Therefore, it is necessary to optimize the combination (such as the rice/soybean (*Glycine max* L. Merr.) intercropping combination) and improve P uptake and utilization in sustainable agriculture.

The crop root system is regarded as the key to underground species interaction; the highly plastic root morphology modulated by the presence of neighboring crops in intercropping systems can enhance crop P acquisition [13,14]. Previous studies have shown that maize (*Zea mays* L.)/faba bean (*Vicia faba* L.) intercropping can increase crop yield and P uptake through a significant increase in root surface area and root dry weight in both maize and faba bean [6]. Moreover, due to competition between species, intercropping may also increase the root length and P uptake of one intercropped crop and decrease that of another intercropped crop [15]. For example, intercropping increases the root length and P uptake of wheat but decreases the root length and P uptake of soybean in the wheat (*Triticum aestivum* L.)/soybean intercropping system [16]. So far, the majority of dominant crops in cereal/legume intercropping systems were cereals; consistent studies have shown that cereal crops tend to benefit from intercropped legume crops [6,17,18,19]. On the other hand, the relationship between root morphology and P uptake in legume-dominated legume/cereal intercropping systems remains unclear.

The roots of different intercropped crops can form symbiotic mycorrhizal networks, which can effectively transfer resources between plants and promote the absorption of nutrients, especially for P [20,21,22,23]. Studies have shown that rice/mung bean (*Vigna radiata* L. Wilczek) intercropping significantly promotes arbuscular mycorrhizal formation, particularly in rice roots. Intercropping improved mycorrhizal formation and increased total P uptake by 57% in rice and 64% in mung beans [24]. Similar results were found in sorghum (*Sorghum bicolor* L.)/poplar (*Populus* L.) and soybean/acacia (*Vachellia farnesiana* L.) intercropping systems [25,26]. Although intercropping may increase arbuscular mycorrhizal fungi (AMF) colonization, AMF colonization within the same intercropping system has asymmetric effects on different intercropped species [11,27]. The benefits of mycorrhizal fungi associations for crops can vary based on their specific nutrient requirements and reliance on mycorrhizal symbiosis [28,29]. In cereal/legume intercropping systems, AMF plays a key role in alleviating P deficiencies to promote the growth of legume crops [11,28,30]. Meanwhile, it has been demonstrated that the intercropped cereals have a higher variation in mycorrhizal network colonization compared to the legume, suggesting that cereals may rely more on mycorrhizal associations for nutrient acquisition, particularly P [29]. The soil P level has been regarded as a key driver of the AMF colonization and root–microbiome interaction in intercropped crops [31]. Generally, AMF colonization can promote crop P uptake under P-limiting conditions, but their contribution usually decreases along with the increase in P supply [32]. Studies have shown that inoculating AMF significantly increases the P uptake of intercropped maize under low P levels but not at high P levels in a maize/faba bean intercropping [30]. AMF can contribute up to 80% of the total P uptake, and the co-application of mineral P fertilizer and AMF inoculation can reduce the recommended phosphate fertilizer by about 20% under specific conditions [33]. Therefore, it is necessary to study the regulatory effects of AMF on P uptake and yield in legume/cereal intercropping systems at different P levels.

Overall, the effect of root morphological traits (root length, root surface area, root volume and root dry weight) and AMF colonization on P uptake in intercropping systems has been reported by few studies; however, the relative contributions of root morphology and AMF colonization on P uptake in legume-dominated legume/cereal intercropping systems are largely unclear. The objectives of this study with field and potted experiments were as follows: (1) to investigate whether rice/soybean intercropping under dry cultivation can improve crop P uptake and biomass at different P levels, and (2) to evaluate the relative contribution of root morphological traits and AMF colonization on the P uptake in a dry-cultivated rice/soybean intercropping system.

## 2. Results

### 2.1. Crop Biomass, Yield Response Efficiency (YRE) and P Uptake

**Figure 1:**
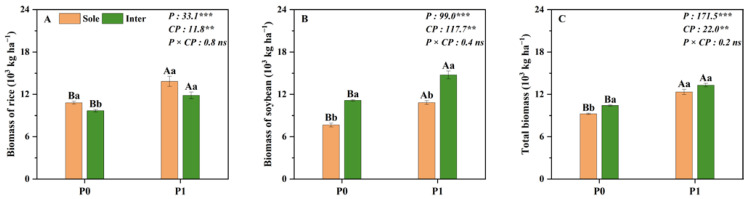
Rice (**A**), soybean (**B**) and total (**C**) biomass under monoculture and intercropping pattern at two P levels in the field. Sole represents monoculture, and Inter represents rice/soybean intercropping system; P0 and P1 represent without and with P fertilizer addition, respectively; P represents P level; CP represents cropping pattern. Different capital letters represent significant differences between two P levels within the same cropping pattern at *p* < 0.05; different lowercase letters denote significant differences between the different cropping patterns within the same P level at *p* < 0.05. Values = means ± SE (*n* = 5). The values are the F values; **, *** and “ns” indicate significance at *p* < 0.01, *p* < 0.001 and no significant difference, respectively.

**Figure 2:**
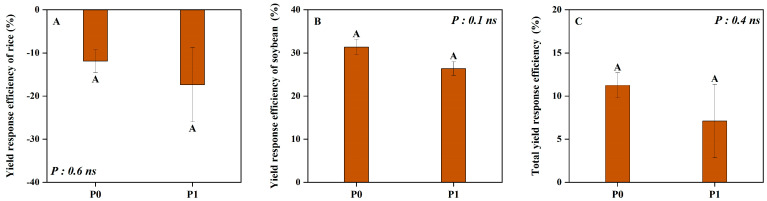
Rice (**A**), soybean (**B**) and total (**C**) yield response efficiency under two P levels in the field. P0 and P1 represent without and with P fertilizer addition, respectively. The same capital letters represent no significant difference between the two P levels at *p* < 0.05. Values = means ± SE (*n* = 5). The values are the F values; “ns” indicates no significant difference between two P levels.

The rice biomass, soybean biomass and total biomass in the field were significantly affected by P level and cropping pattern (Figure 1). Compared to the corresponding monoculture, intercropping significantly reduced the biomass of rice by 14% but appreciably improved that of intercropped soybean by 41%, irrespective of the P levels (Figure 1A,B). Thus, the total biomass of intercropping was 10% higher than the monoculture system with an average of two P levels (Figure 1C). Compared with no P application (P0), P application (P1) significantly increased the rice biomass, soybean biomass and total biomass by 25%, 36% and 31% averaged across the two cropping patterns, respectively. Similarly, regardless of the P level, the YRE of rice was both less than zero, while the YRE of soybean and total yield response efficiency were all greater than zero (Figure 2). Thus, intercropping decreased rice yield, while increasing that of soybean. Furthermore, all the YREs were higher at P0 than at P1, indicating that the increased percentage of intercropping yield compared to monoculture was higher at P0 than at P1 in the field (Figure 2).

**Figure 3:**
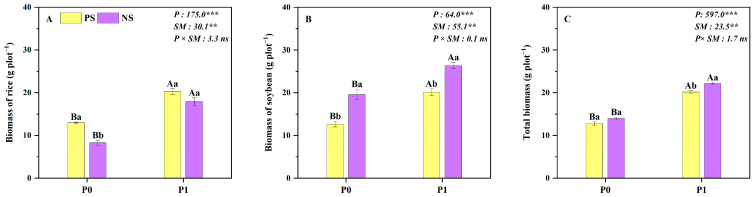
Rice (**A**), soybean (**B**) and total biomass (**C**) under the different root separation modes at two P levels in pots. PS represents a complete plastic root separation between rice and soybean grown in pots; NS represents no root separation between rice and soybean grown in pots. P0 and P1 represent without and with P fertilizer addition, respectively; P represents P level; SM represents root separation mode. Different capital letters represent significant differences between two P levels within the same root separation mode at *p* < 0.05; different lowercase letters denote significant differences between the different root separation modes within the same P level at *p* < 0.05. Values = means ± SE (*n* = 5). The values are the F values; **, *** and “ns” indicate significance at *p* < 0.01, *p* < 0.001 and no significant difference, respectively.

**Figure 4:**
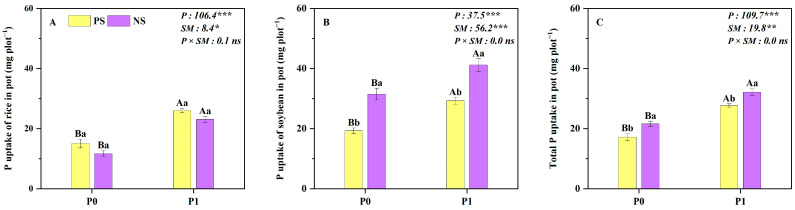
Rice (**A**), soybean (**B**) and total P uptake (**C**) under the different root separation modes at two P levels in pots. PS represents a complete plastic root separation between rice and soybean grown in pots; NS represents no root separation between rice and soybean grown in pots. P0 and P1 represent without and with P fertilizer addition, respectively; P represents P level; SM represents root separation mode. Different capital letters represent significant differences between two P levels within the same root separation mode at *p* < 0.05; different lowercase letters denote significant differences between the different root separation modes within the same P level at *p* < 0.05. Values = means ± SE (*n* = 5). The values are the F values; *, **, *** and “ns” indicate significance at *p* < 0.05, *p* < 0.01, *p* < 0.001 and no significant difference, respectively.

The P level and root separation modes had significant effects on rice, soybean and total biomass, as well as the rice, soybean and total P uptake in pots (Figure 3 and Figure 4). Compared to the complete plastic root separation mode (PS), the no root separation mode (NS) decreased the biomass and P uptake of rice by 27% and 18% (Figure 3A and Figure 4A), while the biomass and P uptake of soybean increased by 40% and 50% (Figure 3B and Figure 4B), respectively. Thus, the total biomass and total P uptake in NS treatment were higher than that in PS treatment by 9% and 20%, respectively (Figure 3C and Figure 4C). Moreover, compared to the no P application, P application increased the rice, soybean and total biomass as well as the P uptake by 35–117% and 31–98% averaged across the two root separation modes. Specifically, P application increased total yield and P uptake by 58% and 61% under monoculture and by 59% and 49% under intercropping, respectively, compared to no P application (Figure 3 and Figure 4).

### 2.2. Root Morphology of Rice and Soybean

**Figure 5:**
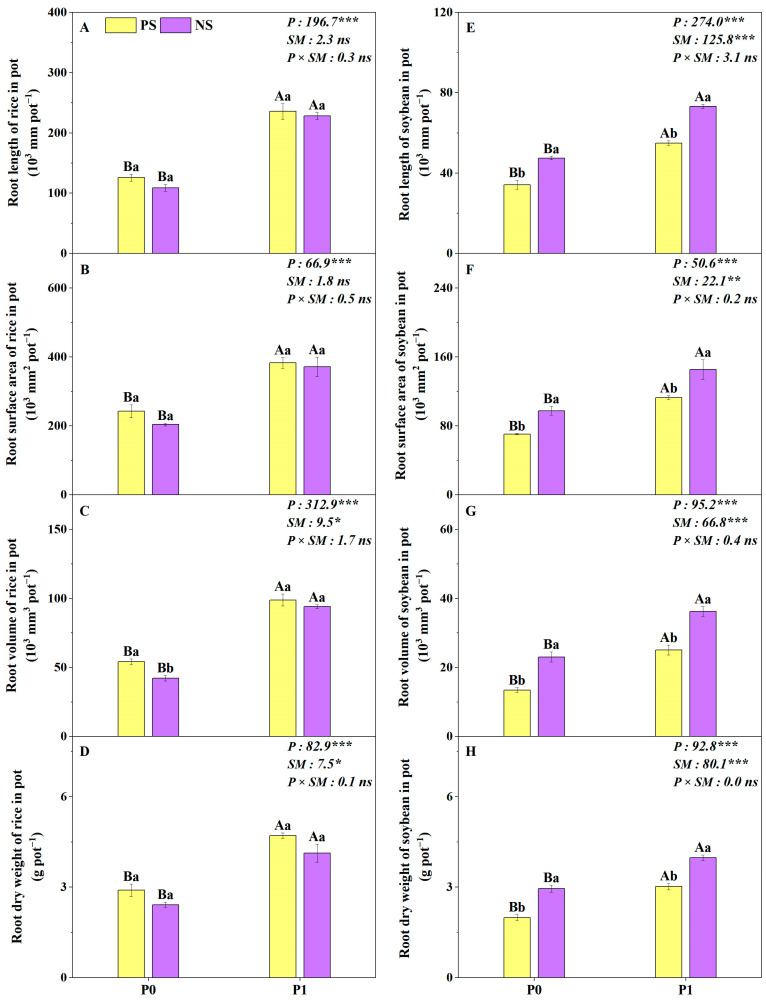
Root length, root surface area, root volume and root dry weight of rice (**A**–**D**) and soybean (**E**–**H**) under the different root separation modes at two P levels in pots. PS represents a complete plastic root separation between rice and soybean grown in pots; NS represents no root separation between rice and soybean grown in pots. P0 and P1 represent without and with P fertilizer addition, respectively; P represents P level; SM represents root separation mode. Different capital letters represent significant differences between two P levels within the same root separation mode at *p* < 0.05; different lowercase letters denote significant differences between the different root separation modes within the same P level at *p* < 0.05. Values = means ± SE (*n* = 5). The values are the F values; *, **, *** and “ns” indicate significance at *p* < 0.05, *p* < 0.01, *p* < 0.001 and no significant difference, respectively.

Root separation modes had a significant impact on the root volume, root dry weight of rice, root length, root surface area, root volume and root dry weight of soybean in the pot experiment (Figure 5C–H). Compared to the PS treatment, the root volume and root dry weight of rice under NS treatment were significantly reduced by 12% and 16%, respectively (Figure 5C,D), while the root length, root surface area, root volume and root dry weight of soybean were increased by 33–54% (Figure 5E–H). In addition, all the measured root parameters of potted rice and soybean were significantly affected by the P levels (Figure 5). Compared to the P0 level, the P1 level significantly increased the root length, root surface area, root volume and root dry weight of rice and soybean by 42–100% averaged across two root separation modes (Figure 5).

### 2.3. Mycorrhizal Infection Density (MID), Arbuscular Mycorrhiza Richness (AMR) and Vesicle Richness (VER) of Rice and Soybean

**Figure 6:**
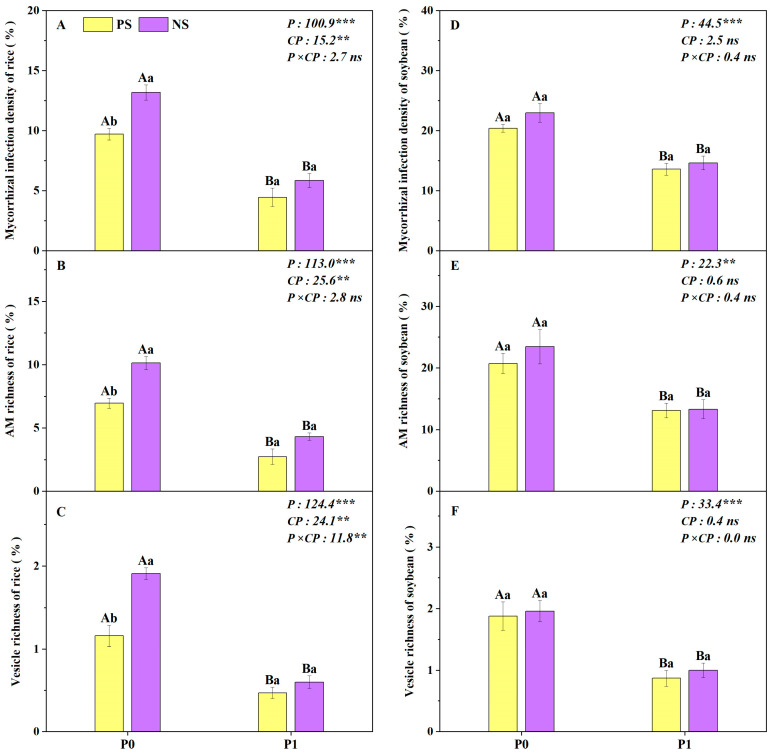
Mycorrhizal infection density, arbuscular mycorrhiza (AM) richness and vesicle richness of rice (**A**–**C**) and soybean (**D**–**F**) under the different root separation modes at two P levels in pots. PS represents a complete plastic root separation between rice and soybean grown in pots; NS represents no root separation between rice and soybean grown in pots. P0 and P1 represent without and with P fertilizer addition, respectively; P represents P level; SM represents root separation mode. Different capital letters represent significant differences between two P levels within the same root separation mode at *p* < 0.05; different lowercase letters denote significant differences between the different root separation modes within the same P level at *p* < 0.05. Values = means ± SE (*n* = 5). The values are the F values; **, *** and “ns” indicate significance at *p* < 0.01, *p* < 0.001 and no significant difference, respectively.

The MID, AMR and VER of potted rice were significantly affected by the P level and root separation modes, and VER was also significantly affected by the interaction between the P level and the root separation mode (Figure 6A–C). At P0 level, the MID, AMR and VER of rice under the NS treatment were significantly higher by 36%, 46% and 65%, respectively, compared to the PS treatment, while there was no significant difference under P1 level (Figure 6A–C). Generally, the MID, AMR and VER of rice under the P0 level were significantly higher than those under the P1 level, regardless of the root separation mode (Figure 6A–C). As for soybean, the P level had significant effects on the MID, AMR and VER of soybean (Figure 6D–F). The MID, AMR and VER of soybean were significantly reduced by P application compared to those without P application (Figure 6D–F).

### 2.4. The Relative Contributions of Root Morphological Traits and AMF Colonization on Crop P Uptake

**Figure 7:**
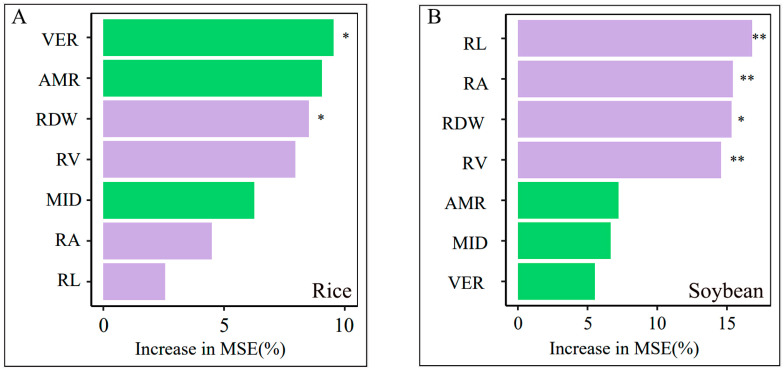
Random forest analysis to identify the main predictors of P uptake in rice (**A**) and soybean (**B**). * and ** indicate significance between the predictors and P uptake at *p* < 0.05 and *p* < 0.01. Abbreviations of the conceptual schema are defined as follows: root length (RL); root surface area (RA); root volume (RV); root dry weight (RDW); mycorrhizal infection density (MID); arbuscular mycorrhiza richness (AMR); vesicle richness (VER); mean square error (MSE).

The random forest analysis consistently identified the VER as exhibiting a higher random forest mean predictor importance in the prediction of P uptake in rice, followed by AMR (Figure 7A). As for soybean, root length was the most important factor affecting the P uptake in intercropping, followed by root surface area and root dry weight (Figure 7B). Thus, the P uptake in soybean was mainly influenced by root morphology, while that of rice was more dependent on the AMF colonization in the rice/soybean intercropping system.

## 3. Discussion

### 3.1. Improved Biomass and P Uptake in Dry-Cultivated Rice/Soybean Intercropping

Cereal/legume intercropping has been regarded as a widely used agricultural system with significant advantages for production and P acquisition, such as the millet (*Panicum miliaceum* L.)/mung bean and maize/chickpea (*Cicer arietinum* L.) intercropping systems [13,19,34,35,36,37]. Consistent results were obtained in both field and pot experiments: total biomass and P uptake were significantly higher in rice/soybean intercropping or no root separation mode compared to the monoculture or root separation mode at both P levels (Figure 1, Figure 3 and Figure 4). The increase in total biomass and P uptake in the intercropping system was mainly due to a larger biomass increase in the intercropped soybean, which compensated for a slight reduction in the intercropped rice biomass (Figure 1). This result can be explained by the asymmetric interspecific competition in the intercropping system that strongly depends on intercropped species [38]. In the rice/soybean intercropping system, soybean exhibits greater competitiveness than rice, enabling it to derive more benefits from the intercropping system, making it the dominant species [14,15]. With regard to rice, it is disadvantaged in the context of intercropping because of more competition than facilitation on cereal crops, resulting in a reduction in biomass and P uptake in the intercropped rice [15,39].

Compared to the no P application condition, our results clearly demonstrated that the total biomass of intercropping was higher with P application (Figure 1 and Figure 3). However, the total yield response efficiency under the P0 level was higher than that under the P application (Figure 2). This may be due to the fact that P application can reduce the contribution of the acid phosphatase activity of root exudates and the carboxylate secretion of intercropped soybean to P uptake in our study (unpublished data), compared to P0, resulting in a weaker facilitation effect in the rice/soybean intercropping system [40]. Similar results were also reported in the maize/alfalfa intercropping system, where the complementary effect decreased along with the N gradient [41]. Additionally, the well-tested ‘stress-gradient hypothesis’ (SGH) proposed by Bertness and Callaway (1994) also predicts that positive interactions (facilitation) increase in response to increasing environmental stress [12]. Therefore, rice/soybean intercropping is particularly valuable in resource-limited situations, such as in low-input agroecosystems.

### 3.2. Contributions of Root Morphology and AMF to the Intercropped Crop P Uptake

The root strategies of P uptake are closely related to root competition between the intercropping species [42]. Previous studies have shown that cereals are usually more competitive than legumes, making cereals the dominant species in the intercropping systems [42,43]. This was mainly due to the fact that intercropping promotes the root length or root surface area of cereal crops compared to monoculture, thus resulting in more P uptake [44]. However, our study revealed that intercropping rice with soybean suppressed the morphological traits of rice, leading to reduced root volume and root dry weight, further limiting the rice P uptake (Figure 5). To cope with this stress, rice may adopt additional strategies beyond root morphological changes to acquire soil P. Through random forest analysis, we further found that AMF colonization contributed more to the P uptake of intercropped rice than root morphology (Figure 7). This indicates that when the root growth of intercropped rice is inhibited, it mainly obtains P from the soil by enhanced AMF colonization [45]. Consistent with previous studies, when crop growth is inhibited, crops can increase the AMF colonization to promote the absorption of water and nutrients by host plants, especially the P from insoluble soil fractions [46,47]. Therefore, our results indicate that interspecific competition can drive the changes in P uptake strategies and activate the nutrient acquisition potential of crops under stressful conditions [48,49,50,51]. A similar result was found in other cereal/legume intercropping systems, where AMF colonization increased in the disadvantaged cereal species of the maize/alfalfa intercropping systems [52]. This indicates that AMF colonization can promote P uptake and utilization by disadvantaged species in the intercropping systems.

The high phenotypic plasticity of root morphology can increase the competitiveness of crops for resources and against neighbors, making them dominant species [53,54]. In our study, intercropped soybeans, due to the higher phenotypic plasticity of root morphology, became the dominant species in the rice/soybean intercropping system (Figure 5). Our results further found that the increase in the P uptake of soybean was mainly attributed to root morphology rather than AMF colonization, where root length contributed the most to the P uptake of intercropped soybean (Figure 7). This indicates that intercropped soybean mainly depends on root length for acquiring soil P rather than AMF colonization. This result is in line with previous studies showing that intercropping promoted the root length and root surface area of soybean, leading to increased P uptake [16,17,18,19]. In these studies, soybean, as the dominant species in the intercropping system, has stronger belowground competitiveness and can compete for water and nutrients in the root zone of the intercropped disadvantaged species of rice [19,55].

A number of studies have proved that intercropping can increase the colonization of AMF in legume crops [11,28,29]. In our study, the differences in the AMF colonization of soybean between intercropping and monoculture were not significant (Figure 6). This is because soybean can directly absorb P from the soil through root morphology; it can also use root exudation to activate the insoluble P in soil to meet the needs of P for its growth [56]. Similar results have been found in other studies: intercropping increased the root surface area and root length of legumes but inhibited the root morphological traits of the cereal in the maize/alfalfa intercropping systems [57,58]. In the present study, intercropped rice and soybean used different root strategies to uptake P from the soil. The mycorrhizal pathway was chosen by the intercropped disadvantaged species, while the root morphology pathway was used by the intercropped dominant species to promote their P uptake and utilization (Figure 5 and Figure 6). In summary, the stronger competitive ability of dominant species increases the root variability between cereals and legumes, and larger differences in root traits may lead to smaller ecological niche overlaps and drive positive complementary effects, thus promoting P uptake and utilization in intercropping systems [59,60,61,62].

### 3.3. The Effects of P Level on the Relative Contribution of Root Morphology and AMF Colonization in Rice/Soybean Intercropping Under Dry Cultivation

The root morphological traits and AMF colonization of crops are closely related to the availability of P in soil [15,63]. In this study, the application of P fertilizer increased the root morphological traits of crops while decreasing the colonization of AMF (Figure 5 and Figure 6). The increase in crop root morphology may be due to the increase in the available P content in soil after the application of P fertilizer, which subsequently promotes plant growth [14,64]. For AMF, the mycorrhizal infection density, AM richness and vesicle richness were significantly increased without P fertilizer application compared to the P fertilizer application condition (Figure 6). This is because when crops are faced with intense interspecific competition and a P-limiting environment, plants will cooperate with AMF in exchange for nutrients needed for growth at the expense of photosynthetic products [51]. After symbiosis between crops and AMF, a huge underground mycorrhizal network can be used to resist environmental stress [52]. In addition, high P availability in the soil reduces the carbon (C) allocated by crops to the roots, thereby directly inhibiting the carbon availability to AMF [63] and shifting the mycorrhizal function from mutualistic to parasitic [65,66]. Therefore, crops will choose different P uptake strategies to meet their P requirements at varying soil P levels. This study fills a gap in research on the relative contribution of AMF colonization or root morphology to P uptake in rice/soybean intercropping under dry cultivation, thereby enriching the knowledge base for sustainable agricultural production.

## 4. Materials and Methods

### 4.1. Field Experiment

The field experiment was conducted in 2023 at the research base of Jilin Agricultural University in Changchun, Jilin Province (43°81′ N, 125°40′ E). The annual mean temperature ranged from 4.6 to 6.4 °C, the precipitation was 465.8 mm (Figure S1), the annual average evaporation was 1500–2000 mm, and the annual accumulated temperature (≥10 °C) was 2546–3375 °C. The soil type was meadow black soil, which was classified as Solonetz, according to the World Reference Base for Soil Resources, and the physical and chemical properties of the 0–20 cm surface soil were as follows: pH of 6.90, organic carbon 26.15 g kg^−1^, total N of 2.03 g kg^−1^, available K of 166.50 mg kg^−1^, and Olsen-P of 21.54 mg kg^−1^.

The field experiment was split into two main plots with crops treated with two P levels: no P fertilizer was applied (P0), and 60 kg P ha^−1^ was continuously applied (P1); the subplot was treated with different cropping patterns with four replications. There were three cropping modes in each block: monoculture rice under dry cultivation (Suijing 18; MR), monoculture soybean (Jinong 89; MS), and rice/soybean intercropping (IRS). Eight rows of rice or soybean crops were planted in the cropping mode of monoculture. The dry-cultivated rice/soybean intercropping system used a 2.4 m intercropping zone consisting of a 1.2 m wide rice zone and a 1.2 m wide soybean zone, with each intercropping plot consisting of three intercropping strips, each consisting of four rice rows and four soybean rows. The row spacing of both crops was 0.3 m, with a length of 6 m, which was the same under monoculture and intercropping cropping modes (Figure S3).

The fertilization levels of the two crops were applied in accordance with the local production of conventional fertilization. For rice, 75 kg ha^−1^ of K_2_O was applied as the base fertilizer at one time, and 160 kg ha^−1^ N fertilizer was applied in a ratio of 5:3:2 as base fertilizer (at sowing), tiller fertilizer (at tillering) and spike fertilizer (at booting), respectively. Soybean was supplied with 75 kg N ha^−1^ and 75 kg K ha^−1^; all fertilizers were broadcast to the soil surface at sowing as base fertilizer. The fertilizers used in the experiment were urea (N content 46%), superphosphate (P_2_O_5_ content 12%) and potassium chloride (K_2_O content 60%). The experimental crops were sown in early May and harvested in early October. Both rice and soybean were grown under rain-fed conditions throughout the growth period. When the soil water potential of 10–15 cm depth is lower than −35 kPa, irrigation is carried out, and the soil water potential reaches −10 kPa after watering. Soil water potential was monitored twice a week using a soil water potential meter (SYS-TSS1; Sayas Technology Co., Ltd., Shenyang, China). Weeds, diseases and pests were managed as needed during the growing season.

### 4.2. Pot Experiment

**Figure 8:**
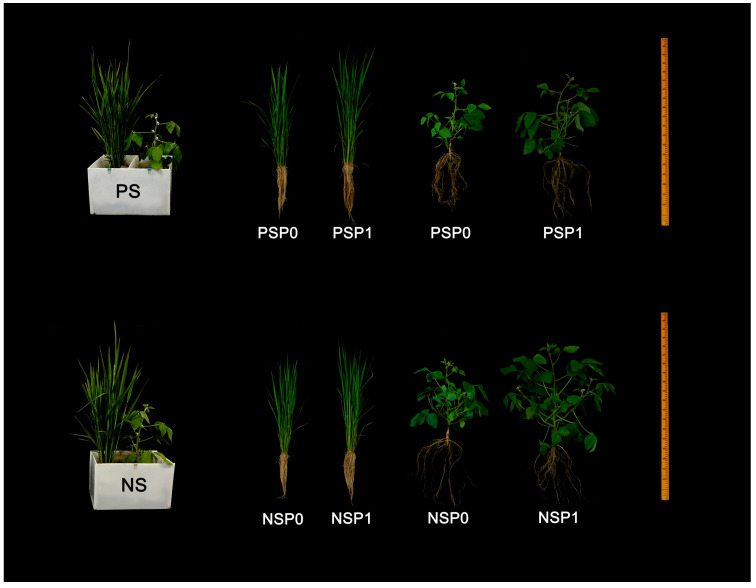
Schematic diagram showing the root separation modes in pots and plant performance of rice and soybean at harvest time. PS represents a complete plastic root separation between rice and soybean grown in pots; NS represents no root separation between rice and soybean grown in pots. P0 and P1 represent without and with P fertilizer addition, respectively.

In the same year as the field experiment, a pot experiment was conducted in a greenhouse at Jilin Agricultural University. A two-factor randomized block design was used in the pot experiment, and treatments included two P levels: no P (P0) and 60 kg ha^−1^ P (P1); two root separation modes were set up: with complete plastic root separation (PS) and with no root separation (NS; Figure 8), with 5 replicates. The PS treatment eliminates interspecific root interactions, while the NS treatment allows for interspecific root promotion and competition; similar pot experiments were also used in other studies [67,68]. Using indigenous AMF as inoculum, soil was collected from 0 to 20 cm in field experiment site. The presence of AMF inoculants was assessed by wet sieving [69], followed by observation of AMF spores and mycelial fragments under a stereomicroscope. AMF inoculants include roots, spores and mycelium naturally occurring in soil. The soil was thoroughly mixed, and pebbles and other debris were discharged. This unsterilized soil was put into each plastic basin (40 cm long, 20 cm wide and 25 cm high). The physical and chemical properties of the soil were recorded at the beginning of the experiment, with pH of 6.46, organic carbon content of 13.42 g kg^−1^, total N of 1.48 g kg^−1^, available K of 22.82 mg kg^−1^, and Olsen-P of 28.02 mg kg^−1^.

On 14 October 2023, rice under dry cultivation was planted at a depth of 2 cm in the soil of the container with 5 seeds per hole, and soybeans were planted at a depth of 3 cm in the soil of the container with 1 seed per pot. Each species in the pot occupied half of the soil area following the same fertilizer application strategies as described in the field experiment. During the experiment, weeds, diseases and pests were regularly removed, and water management was carried out as in the field experiment. Greenhouses are transparent, and crops grow by natural light. Crops were harvested after 70 days of planting (booting stage).

### 4.3. Sampling and Determination

#### 4.3.1. Biomass and Grain Yield

In the field experiment, crop stalk and grain were sampled separately for biomass and grain yield determination at physiological maturity (early October). In monoculture systems, three middle rows of rice or soybean were sampled. In the second strip of intercropping systems, rice or soybean in the first two rows bordering another intercropped species was harvested for sampling. The aboveground biomass was measured by threshing and air drying in the field. In the potted experiment, after harvesting, the complete above-ground parts were taken and dried; then, their biomass was weighed and recorded.

#### 4.3.2. Root Morphological, AM Root Colonization and P Uptake

After pot harvest, the roots of each plant were thoroughly cleaned with water manually, and the roots were randomly divided into two subsamples: (i) for analysis of root morphological traits, and (ii) for analysis of root colonization by AM fungi (first-order root). All root subsamples were stored in 50% ethanol at 4 °C until analysis.

The samples for root morphology analysis were analyzed using a flatbed scanner (Perfercion V700 Photo; Epson America Co., Ltd., Los Alamitos, CA, USA) at a resolution of 23.6 pixels mm^−1^ (600 dpi) and then analyzed using the image processing software WinRhizo Pro 2013 (Regent Instruments, Québec, QC, Canada) to determine the total root length, root surface area and root volume. The roots and their above-ground parts were then dried at 65 °C for 72 h, and the dry weight was recorded. The dried stems were crushed to determine the P content, and the crop P content was determined by the molybdenum vanadium phosphate method.

Root subsamples for AMF colonization analysis were cut into 1 cm pieces and dispersed in water. Root pieces were randomly selected and cleared with 10% KOH, stained with 0.05% trypan blue, and stored in lactoglycerol (1:1:1) [70]. To determine the AMF colonization rate, we used the modified intersections method [71]. Thirty root pieces (1 cm in length) were observed at × 200 total magnification with a compound microscope. One hundred intersections of each sample were examined for the presence or absence of AMF mycelia, vesicles, or arbuscles. The arbuscular mycorrhizal infection density (MID), arbuscular mycorrhiza richness (AMR) and vesicle richness (VER) of each root segment were calculated using the MYCOCALC software (www2.dijon.inra.fr/mychintec/Mycocalc-prg/download) [72].

### 4.4. Calculations

The total biomass of the intercropping system can be compared with the weighted average of the corresponding monoculture crops based on their proportions of the area in the intercropping system [4]. The weighted average of total biomass is calculated as follows:

Monocultured biomass = RB_m_ × PR + SB_m_ × PS (1)

Intercropping biomass = RB_i_ × PR + SB_i_ × PS (2)

where RB_m_ and RB_i_ represent rice biomass under monoculture and intercropping, respectively; SB_m_ and SB_i_ represent soybean biomass under monoculture and intercropping, respectively; and PR and PS represent the area proportion of rice and soybean in intercropping system, respectively. The above formula is also used to calculate the weighted average of grain yield and P uptake.

We also calculated the yield response efficiency (YRE) and the response efficiency of intercropping relative to monoculture cropping mode as follows [73]:

YRE = (Y_i_ − Y_m_)/Y_m_ × 100% (3)

where Y_m_ and Y_i_ represent the grain yield of the same crop under monoculture and intercropping, respectively.

### 4.5. Statistical Analysis

The normal distribution and homogeneous variance of all data were tested using the Shapiro–Wilk test in SPSS 22.0 software (SPSS, Inc., Chicago, IL, USA). Two-way analysis of variance (P level and cropping pattern in the field experiment and P level and root separation mode in the pot experiment) was used to evaluate the effects of P level and crop pattern or root separation mode on each index. When interactions were significant, one-way ANOVA was used to assess the effect of a single factor by fixing another factor, and Duncan’s multiple comparison range test was used to compare significant differences in the means. The significance level was set as *p* < 0.05. Random forest analysis was used to further reveal the relative contribution of root morphological traits and AMF colonization to P uptake of rice and soybean.

## 5. Conclusions

The implementation of dry-cultivated rice/soybean intercropping enhanced the total biomass and P uptake, with P fertilization also amplifying these benefits. The intercropping advantage was primarily driven by increased biomass and P uptake in the intercropped soybean, which offset a slight reduction in these parameters for intercropped rice. In this system, soybean, as the dominant species, primarily relied on the modification of root morphological traits for P acquisition, while the less competitive rice depended on AMF colonization for P uptake. These findings suggest that intercropped species adopt distinct P acquisition strategies to meet their requirements. This study provides insights into optimizing P fertilizer management by balancing root morphological plasticity and AMF colonization in the legume-dominated legume/cereal intercropping systems.

## Data Availability

Data is contained within the article and Supplementary Materials.

## Supplementary Materials

The following supporting information can be downloaded at: https://www.mdpi.com/article/10.3390/plants14010106/s1, Figure S1: Geographical location map for field experiment site; Figure S2: The precipitation (bar) and mean air temperature (curve) during the growth season in 2023 at the field site; Figure S3: Actual field photographs of the three cropping 26 systems in this study. Monoculture rice (MR), monoculture soybean (MS), rice/soybean intercropping (IRS) with alternating strips of four rows of rice and four rows of soybean.
