# Supplementary material for: The Relative Contribution of Root Morphology and Arbuscular Mycorrhizal Fungal Colonization on Phosphorus Uptake in Rice/Soybean Intercropping Under Dry Cultivation
Source: Plants (Basel). 2025 Jan 2;14(1):106. doi: 10.3390/plants14010106 (PMC11723421; doi:10.3390/plants14010106)
Supplement: Supplementary file 1 [file plants-14-00106-s001.zip › plants-3352006-supplementary.pdf]

# Supplementary Materials for

**The relative contribution of root morphology and arbuscular mycorrhizal fungal colonization on P uptake of dry-cultivation rice/soybean intercropping system**

Huimin Ma, Hongcheng Zhang, Qian Gao, Shilin Li, Yuanyuan Yu, Jiaying Ma, Congcong Zheng, Meng Cui, Zhihai Wu, Hualiang Zhang

Correspondence author: wuzhihai@jlau.edu.cn; hualiangzhang@zju.edu.cn

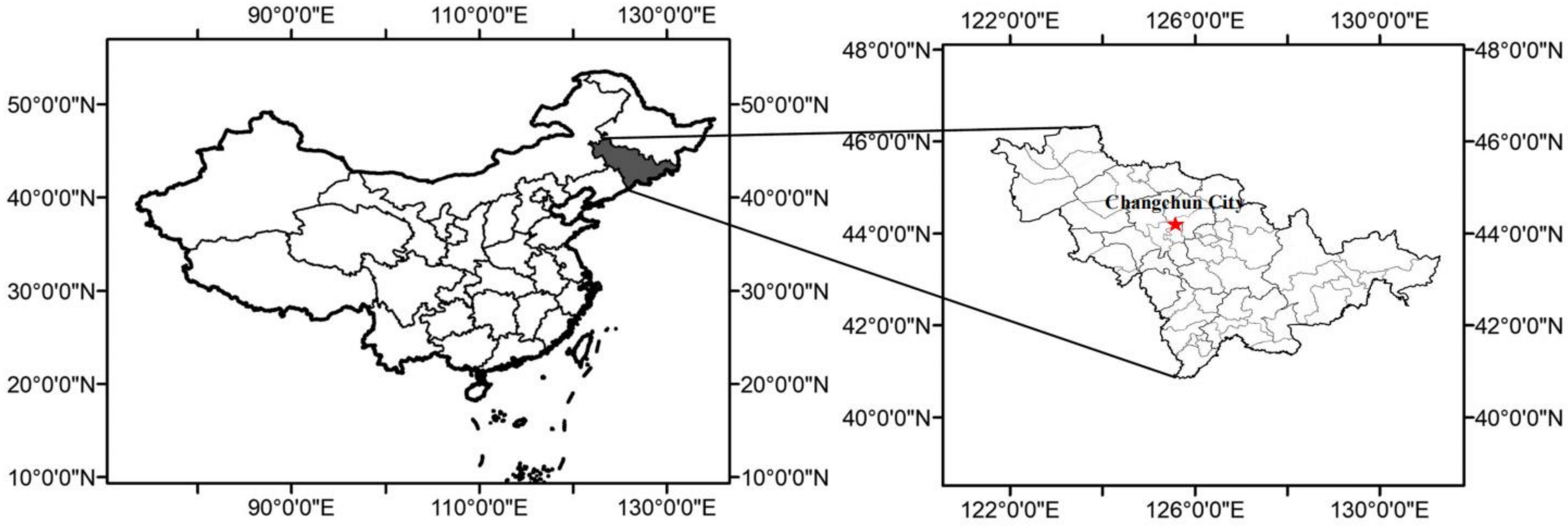


**Figure S1. Geographical location of field experiment**.

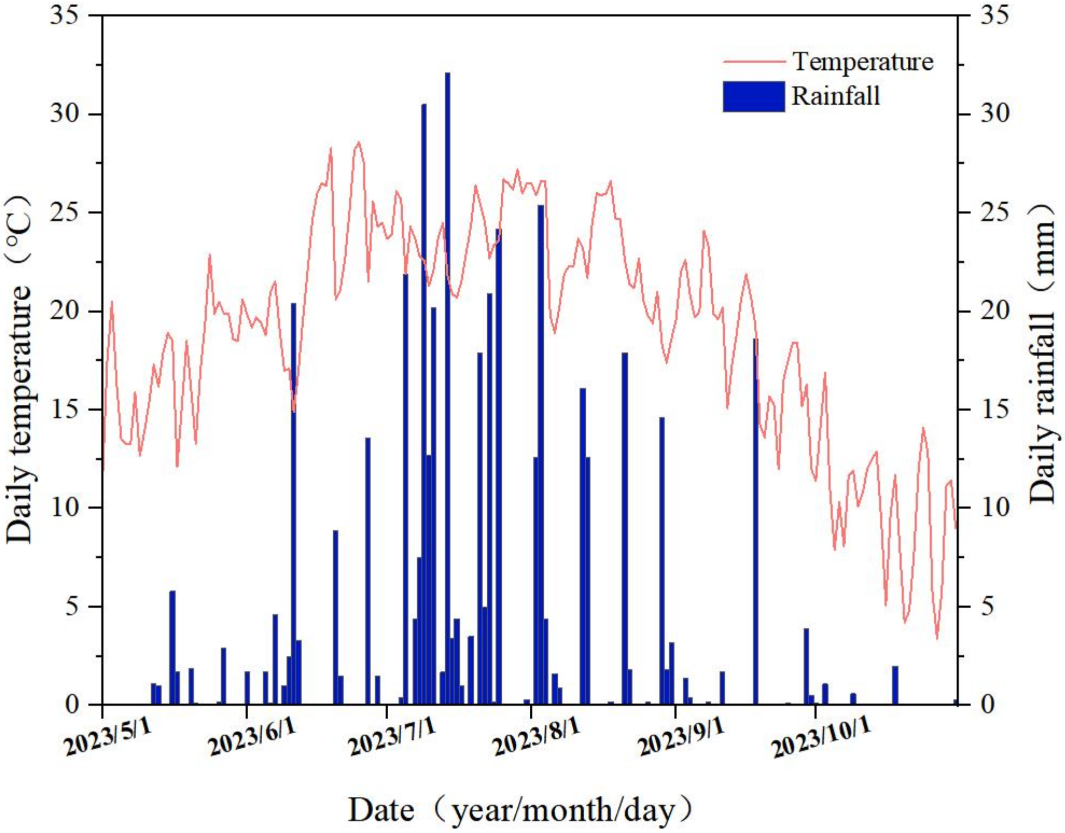


**Figure S2 The precipitation (bar) and mean air temperature (curve) during the growth season in 2023 at the field site.**

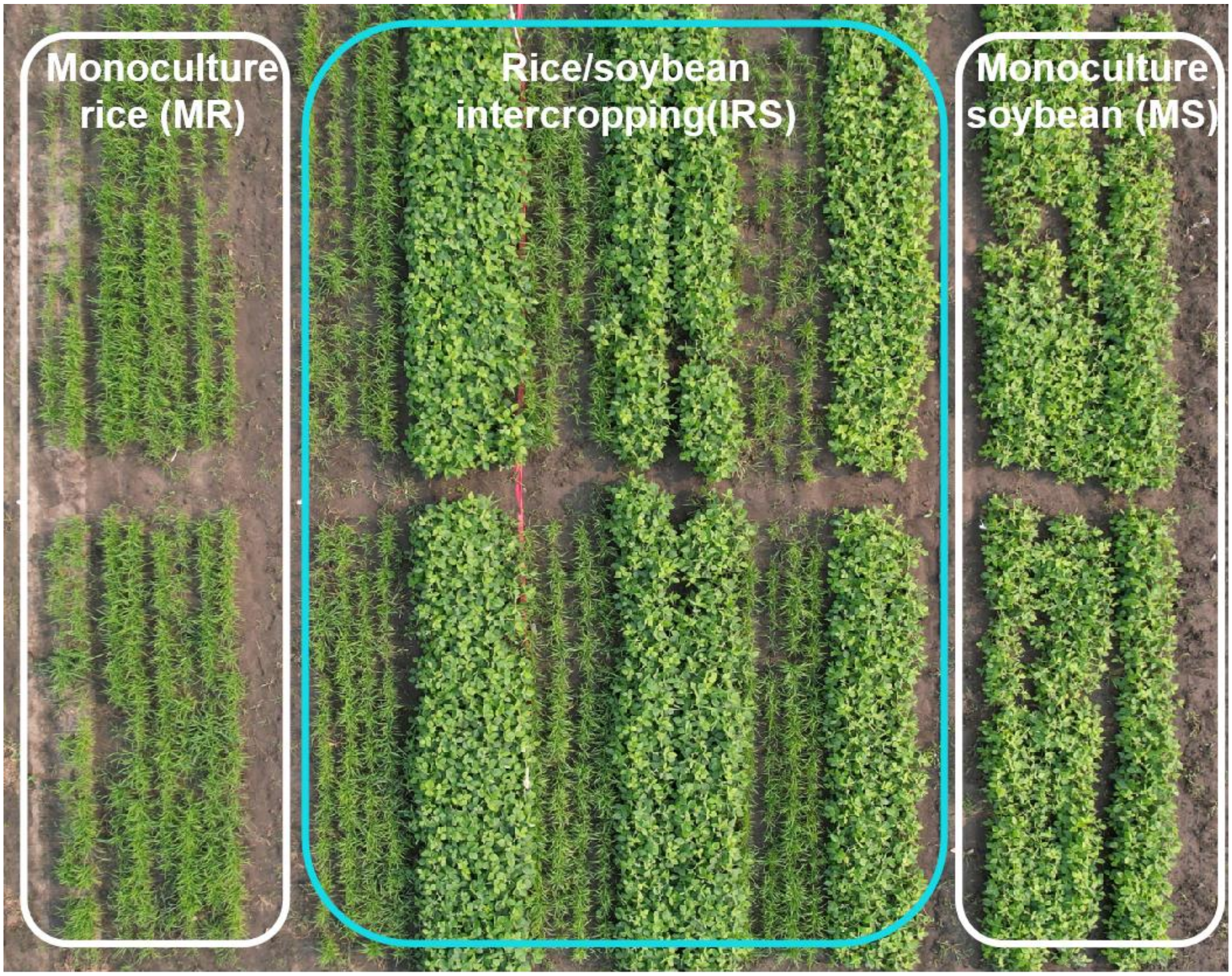


**Figure S3. Actual field photographs of the three cropping systems in this study.** Monoculture rice (MR), monoculture soybean (MS), rice/soybean intercropping (IRS) with alternating strips of four rows of rice and four rows of soybean.
